# Outcomes by *Candida* spp. in the ReSTORE Phase 3 trial of rezafungin versus caspofungin for candidemia and/or invasive candidiasis

**DOI:** 10.1128/aac.01584-23

**Published:** 2024-03-25

**Authors:** Jeffrey B. Locke, Chris M. Pillar, Mariana Castanheira, Cecilia G. Carvalhaes, David Andes, Jalal A. Aram, Christina Andrzejewski, Ken Bartizal, Anita F. Das, Taylor Sandison, George R. Thompson, Peter G. Pappas

**Affiliations:** 1 Cidara Therapeutics, Inc., San Diego, California, USA; 2 Science and Operations, Microbiologics, Kalamazoo, Michigan, USA; 3 JMI Laboratories, North Liberty, Iowa, USA; 4 Department of Medicine, University of Wisconsin-Madison, Madison, Wisconsin, USA; 5 Medical Affairs, Melinta Therapeutics, LLC, Parsippany, New Jersey, USA; 6 Division of Infectious Diseases, Department of Internal Medicine and Department of Medical Microbiology and Immunology, University of California Davis Medical Center, Sacramento, California, USA; 7 Division of Infectious Diseases, Department of Internal Medicine, University of Alabama at Birmingham, Birmingham, Alabama, USA; University Children's Hospital Münster, Münster, Germany

**Keywords:** candidemia, echinocandin, invasive candidiasis, *Candida *species, antifungal therapy

## Abstract

Rezafungin is a long-acting, intravenously administered echinocandin for the treatment of candidemia and invasive candidiasis (IC). Non-inferiority of rezafungin vs caspofungin for the treatment of adults with candidemia and/or IC was demonstrated in the Phase 3 ReSTORE study based on the primary endpoints of day 14 global cure and 30-day all-cause mortality. Here, an analysis of ReSTORE data evaluating efficacy outcomes by baseline *Candida* species is described. Susceptibility testing was performed for *Candida* species using the Clinical and Laboratory Standards Institute reference broth microdilution method. There were 93 patients in the modified intent-to-treat population who received rezafungin; 94 received caspofungin. Baseline *Candida* species distribution was similar in the two treatment groups; *C. albicans* (occurring in 41.9% and 42.6% of patients in the rezafungin and caspofungin groups, respectively), *C. glabrata* (25.8% and 26.6%), and *C. tropicalis* (21.5% and 18.1%) were the most common pathogens. Rates of global cure and mycological eradication at day 14 and day 30 all-cause mortality by *Candida* species were comparable in the rezafungin and caspofungin treatment groups and did not appear to be impacted by minimal inhibitory concentration (MIC) values for either rezafungin or caspofungin. Two patients had baseline isolates with non-susceptible MIC values (both in the rezafungin group: one non-susceptible to rezafungin and one to caspofungin, classified as intermediate); both were candidemia-only patients in whom rezafungin treatment was successful based on the day 30 all-cause mortality endpoint. This analysis of ReSTORE demonstrated the efficacy of rezafungin for candidemia and IC in patients infected with a variety of *Candida* species.

## INTRODUCTION

Commensal *Candida* species (spp.) are present on the skin and mucosa of 50%–70% of healthy individuals, but invasive candidiasis (IC), encompassing candidemia and infection of deep tissues, can occur as an opportunistic infection in immunocompromised or immunosuppressed individuals (1, 2). In healthcare settings, IC and candidemia are among the most frequently seen fungal diseases and bloodstream infections, respectively, and are associated with substantial morbidity and mortality (1–3). Five spp. account for most cases*—C. albicans*, *Nakaseomyces glabratus* (the previous classification as *C. glabrata* is retained in this report), *Pichia kudriavzevii* (termed *C. krusei* herein), *C. tropicalis*, and *C. parapsilosis*—although *C. auris* has also demonstrated high potential for nosocomial transmission since its emergence (1, 2).

IC is treated with systemic antifungals comprising azoles, amphotericin B, and echinocandins, with guidelines recommending echinocandin therapy for first-line use (4, 5). Echinocandins target 1,3-β-D-glucan synthase, resulting in destabilization of the fungal cell wall; however, mutations within *FKS* gene “hotspot” (HS) regions give rise to resistance (6). There has also been a rise in IC cases caused by non-*albicans Candida* spp., such as *C. glabrata* and *C. auris*, which have a higher intrinsic resistance potential (7). Increased rates of fluconazole resistance have also been reported, with intrinsic resistance seen in *C. krusei* (8, 9). As such, there is an urgent need for novel, effective antifungal agents.

Rezafungin received US Food and Drug Administration (FDA) approval in March 2023 for the treatment of candidemia and IC in patients aged ≥18 years with limited or no alternative treatment options (10) and was approved in the European Union for the treatment of IC in adults in December 2023 (11). This next-generation echinocandin is also in development for the prevention of invasive fungal diseases caused by *Candida*, *Aspergillus*, and *Pneumocystis* spp. in patients undergoing allogeneic blood and marrow transplantation (12). Compared with other echinocandins, rezafungin has an increased molecular stability, which results in a longer half-life that translates to higher, front-loaded exposure and allows weekly, rather than daily, administration (6, 13). The front-loaded exposure enhances antifungal activity early in treatment, potentially reducing the opportunity for *FKS* resistance-conferring mutations to arise (6, 13). Rezafungin has shown activity against a broad range of isolates, including azole-resistant *Candida* spp., and requires lower pharmacokinetic/pharmacodynamic (PK/PD) target exposures than other echinocandins (14, 15), which could enable treatment of some isolates with elevated MICs (13, 14). A study using 2021 provisional Clinical and Laboratory Standards Institute (CLSI) clinical susceptible-only breakpoints showed that a global 2019–2020 panel of *Candida* spp. had high susceptibility to rezafungin based on MICs (16). The inclusion of a CLSI MIC susceptibility breakpoint for rezafungin against *C. auris* (≤0.5 µg/mL) in the most recent CLSI Performance Standards for Antifungal Susceptibility Testing of Yeasts document (the provisional CLSI breakpoints were approved as of 20 January 2024, unpublished data) also highlights the promise of rezafungin, given this is the first *C. auris* susceptibility breakpoint defined for an antifungal agent (17).

The Phase 3 ReSTORE trial in patients with candidemia and/or IC, on which FDA approval was based, compared weekly treatment with rezafungin vs daily treatment with caspofungin, an established echinocandin (12). Rezafungin was non-inferior to caspofungin for the efficacy endpoints of all-cause mortality at day 30 (primary endpoint for FDA) and global cure at day 14 (primary endpoint for European Medicines Agency), with a similar safety profile. Here, we report an analysis of efficacy outcomes from the ReSTORE study in subgroups defined by baseline pathogen and susceptibility. Isolates demonstrating reduced echinocandin susceptibility were characterized for the presence of *FKS* mutations, and findings were evaluated in light of CLSI breakpoints and those breakpoints recently granted by the FDA (18).

## RESULTS

### Patients

The disposition of patients in ReSTORE was reported previously (12). Briefly, 222 patients were screened, and 199 were randomized to receive treatment (100 to rezafungin and 99 to caspofungin). Nine patients did not have a documented *Candida* infection in the 96 hours prior to randomization (*n* = 5 in the rezafungin group; *n* = 4 in the caspofungin group), and three did not receive study drug (two assigned to the rezafungin group, and one to caspofungin). The modified intent-to-treat (mITT) population, therefore, consisted of 187 patients (*n* = 93 patients in the rezafungin arm; *n* = 94 patients in the caspofungin arm) from whom 204 isolates were submitted for identification and susceptibility testing.

#### Baseline Candida spp.

**Fig 1:**
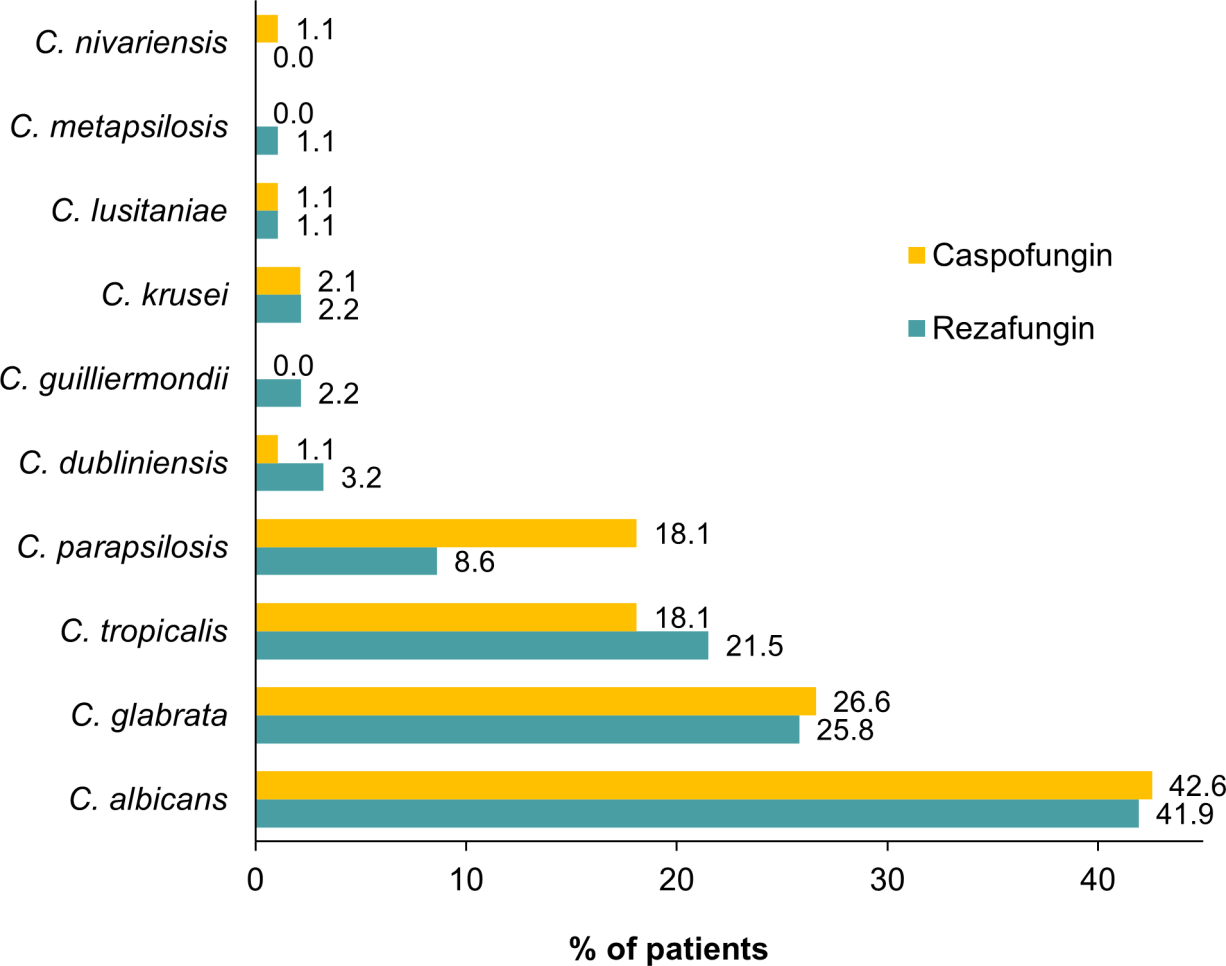
Baseline *Candida* spp. pathogens in the rezafungin and caspofungin treatment groups (mITT population). Data were calculated from 204 identified infections in 187 patients (rezafungin, *n* = 93; caspofungin, *n* = 94). All unique pathogens from the cultures collected within 96 hours prior to randomization or prior to the first dose of the study drug after randomization were summarized. Seventeen patients had multiple isolates at baseline: rezafungin treatment group (no. of patients): *C. glabrata* and *C. tropicalis* (3); *C. albicans* and *C. glabrata* (1); *Candida dubliniensis* and *C. glabrata* (1), *Candida guilliermondii* and *C. tropicalis* (1); *C. parapsilosis* and *C. tropicalis* (1); caspofungin treatment group: *C. albicans* and *C. glabrata* (3); *C. albicans* and *C. tropicalis* (3); *C. glabrata* and *C. tropicalis* (2); *C. albicans* and *C. dubliniensis* (1); *C. glabrata* and *C. krusei* (1).

Distribution of *Candida* spp. at baseline in the rezafungin and caspofungin treatment groups (Fig. 1) was well balanced, with the exception of *C. parapsilosis*, which was detected in 8.6% (8/93) of patients in the rezafungin group and 18.1% (17/94) of patients in the caspofungin group. MIC breakpoints for rezafungin and caspofungin by *Candida* pathogen are reported in Table 1, along with those for anidulafungin and micafungin for additional context. FDA MIC breakpoints for rezafungin are also listed in Table 1 (18). These breakpoints were defined based primarily on clinical and mycological data, with less emphasis on PK/PD, and are, therefore, more conservative than the CLSI values.

**TABLE 1:** Echinocandin MIC breakpoints

*Candida* spp.	Breakpoint MIC (µg/mL)												
	FDA^*a*^	CLSI^*b*^											
		Rezafungin			Anidulafungin			Micafungin			Caspofungin		
	S	S	I	R	S	I	R	S	I	R	S	I	R
*C. albicans*	≤0.12	≤0.25			≤0.25	0.5	≥1	≤0.25	0.5	≥1	≤0.25	0.5	≥1
*C. glabrata*	≤0.12	≤0.5			≤0.12	0.25	≥0.5	≤0.06	0.12	≥0.25	≤0.12	0.25	≥0.5
*C. tropicalis*	≤0.12	≤0.25			≤0.25	0.5	≥1	≤0.25	0.5	≥1	≤0.25	0.5	≥1
*C. parapsilosis*	≤2	≤2			≤2	4	≥8	≤2	4	≥8	≤2	4	≥8
*C. krusei*		≤0.25			≤0.25	0.5	≥1	≤0.25	0.5	≥1	≤0.25	0.5	≥1
*C. guilliermondii*					≤2	4	≥8	≤2	4	≥8	≤2	4	≥8
*C. dubliniensis*		≤0.12											
*C. auris*		≤0.5											

^*a*^ Current FDA-approved breakpoints (18).

^*b*^ CLSI 2022 (17); rezafungin breakpoints are approved by CLSI as of 20 January 2024. I, intermediate; R, resistant; S, susceptible.

#### Fluconazole susceptibility

Although susceptibility to fluconazole was not a focus of the ReSTORE microbiological analyses, MIC values were generated for all isolates. The rate of fluconazole resistance at baseline was comparable between the rezafungin and caspofungin treatment groups. Pooled baseline isolate resistance rates were 0% (0/78) for *C. albicans*, 8.2% (4/49) for *C. glabrata*, 19.4% (7/36) for *C. tropicalis*, and 25.0% (6/24) for *C. parapsilosis*.

### Efficacy outcomes

#### Global cure and mycological eradication

Global cure and mycological eradication at day 14 and treatment-specific MIC values by pathogen spp. at baseline for rezafungin and caspofungin are summarized in Table 2, with the antimicrobial activity against baseline *Candida* spp. detailed in Table S1. Rates of global cure and mycological eradication at day 14 were generally comparable between the two treatment groups.

**TABLE 2:** Global cure and mycological eradication at day 14 by baseline *Candida* species

*Candida* spp.	Characteristic	Rezafungin (400/200 mg)	Caspofungin (70/50 mg)
***C. albicans***	Global cure at day 14, *n*/*N^a^* (%)	21/39 (53.8)	23/40 (57.5)
	Mycological eradication at day 14, *n*/*N^a^* (%)	23/39 (59.0)	24/40 (60.0)
	MIC_90_^*b*^ (MIC range), μg/mL	0.06 (0.008–0.12)	0.06 (0.008–0.12)
	*n^c^*	39	39
***C. glabrata***	Global cure at day 14, *n*/*N^a^* (%)	16/24 (66.7)	14/25 (56.0)
	Mycological eradication at day 14, *n*/*N^a^* (%)	20/24 (83.3)	15/25 (60.0)
	MIC_90_^*b*^ (MIC range), μg/mL	0.12 (0.03–0.5)	0.06 (0.03–0.12)
	*n^c^*	24	25
***C. tropicalis***	Global cure at day 14, *n*/*N^a^* (%)	14/20 (70.0)	10/17 (58.8)
	Mycological eradication at day 14, *n*/*N^a^* (%)	15/20 (75.0)	10/17 (58.8)
	MIC_90_^*b*^ (MIC range), μg/mL	0.06 (0.015–0.12)	0.06 (0.015–0.12)
	*n^c^*	20	16
***C. parapsilosis***	Global cure at day 14, *n*/*N^a^* (%)	6/8 (75.0)	11/17 (64.7)
	Mycological eradication at day 14, *n*/*N^a^* (%)	6/8 (75.0)	14/17 (82.4)
	MIC_90_^*b*^ (MIC range), μg/mL	N/A (0.5–2)	0.5 (0.25–0.5)
	*n^c^*	8	16

^*a*^ n/N is the number of patients with the listed response at day 14 per number of patients with the corresponding *Candida* pathogen at baseline.

^*b*^ For pathogens isolated ≥10 times in a treatment group.

^*c*^ Number of patients with baseline pathogens and susceptibility data available. MIC_90_, minimal inhibitory concentration required to inhibit 90% of isolates tested; N/A, not available.

Treatment-specific MIC_90_ values were also comparable between the two treatment groups (within a twofold difference) for all baseline *Candida* spp. where values could be determined (note, for *C. parapsilosis*, the MIC_90_ for rezafungin could not be estimated due to the small number of isolates; Table 2). Outcomes at day 14 by pathogen spp. and by rezafungin and caspofungin treatment-specific MIC values are given in Table 3. There was no trend for increasing MICs affecting day 14 mycological outcomes for either rezafungin or caspofungin for any of the *Candida* spp.

**TABLE 3:** Outcomes at day 14 by baseline *Candida* spp. and rezafungin and caspofungin CLSI MIC values

*Candida* spp. Treatment (isolates^*b*^)	*n*/*N* (%) by treatment-specific MIC value, µg/mL^*a*^								
	0.008	0.015	0.03	0.06	0.12	0.25	0.5	1	2
*C. albicans*									
Rezafungin [39]									
Global cure	4/7 (57.1)	11/20 (55.0)	4/6 (66.7)	1/4 (25.0)	1/2 (50.0)				
Mycological eradication	5/7 (71.4)	11/20 (55.0)	5/6 (83.3)	1/4 (25.0)	1/2 (50.0)				
Caspofungin [39]									
Global cure	1/2 (50.0)	5/9 (55.6)	11/21 (52.4)	5/6 (83.3)	0/1 (0)				
Mycological eradication	1/2 (50.0)	5/9 (55.6)	12/21 (57.1)	5/6 (83.3)	0/1 (0)				
*C. glabrata*									
Rezafungin [24]									
Global cure			6/8 (75.0)	4/6 (66.7)	6/9 (66.7)		0/1 (0)		
Mycological eradication			7/8 (87.5)	6/6 (100)	6/9 (66.7)		1/1 (100)		
Caspofungin [25]									
Global cure			2/5 (40.0)	11/19 (57.9)	1/1 (100)				
Mycological eradication			2/5 (40.0)	12/19 (63.2)	1/1 (100)				
*C. tropicalis*									
Rezafungin [20]									
Global cure		3/3 (100)	5/8 (62.5)	5/7 (71.4)	1/2 (50.0)				
Mycological eradication		3/3 (100)	5/8 (62.5)	5/7 (71.4)	2/2 (100)				
Caspofungin [16]									
Global cure		1/1 (100)	3/7 (42.9)	4/7 (57.1)	1/1 (100)				
Mycological eradication		0/1 (0)	4/7 (57.1)	4/7 (57.1)	1/1 (100)				
*C. parapsilosis*									
Rezafungin [8]									
Global cure							1/1 (100)	2/4 (50.0)	3/3 (100)
Mycological eradication							1/1 (100)	2/4 (50.0)	3/3 (100)
Caspofungin [16]									
Global cure						5/8 (62.5)	6/8 (75.0)		
Mycological eradication						5/8 (62.5)	8/8 (100)		

^*a*^ *n*/*N* is the number of patients with the listed response at day 14 per number of patients with the corresponding *Candida* pathogen and MIC value at baseline; not all isolates had MIC data.

^*b*^ Number of isolates.

#### All-cause mortality

The observed pattern of day 30 all-cause mortality by baseline pathogen spp. was broadly similar across the rezafungin and caspofungin treatment groups, with most patients who died having *C. albicans*, *C. glabrata*, *C. tropicalis*, or *C. parapsilosis* isolates at baseline (Table 4). Day 30 all-cause mortality and treatment-specific MIC values by baseline pathogen spp. for each group are presented in Table S2.

**TABLE 4:** Day 30 all-cause mortality by baseline *Candida* species (mITT population)

*Candida* spp.	Rezafungin (400/200 mg; *N* = 93)		Caspofungin (70/50 mg; *N* = 94)	
	*n*/*N*1^*a*^	Day 30 ACM, %	*n*/*N*1^*a*^	Day 30 ACM, %
*C. albicans*	11/39	28.2	9/40	22.5
*C. glabrata*	4/24	16.7	2/25	8.0
*C. tropicalis*	5/20	25.0	4/17	23.5
*C. parapsilosis*	1/8	12.5	6/17	35.3
*C. krusei*	1/2	50.0	0/2	0
*C. guilliermondii*	0/2	0	0/0	0
*C. dubliniensis*	0/3	0	0/1	0
*C. lusitaniae*	0/1	0	0/1	0
*C. metapsilosis*	0/1	0	0/0	0
*C. nivariensis*	0/0	0	0/1	0

^*a*^ *n*/*N*1 is the number of patients with the corresponding *Candida* pathogen who died on or before day 30, or with unknown survival status/the number of patients with the corresponding *Candida* pathogen at baseline. ACM, all-cause mortality.

#### Clinical outcomes in patients with baseline isolates that were non-susceptible to rezafungin or caspofungin

Baseline isolates identified in ReSTORE were largely wild-type (WT) and susceptible to rezafungin and caspofungin (based on CLSI interpretation). Breakpoint data are available from two patients (in the rezafungin treatment group) who had baseline isolates with echinocandin non-susceptible MIC values in ReSTORE. One had a *C. dubliniensis* isolate that was non-susceptible to rezafungin, and the other had a *C. glabrata* isolate that was classed as intermediate for caspofungin but was susceptible to rezafungin. Clinical outcomes for these two patients are presented in Table 5. Both were candidemia-only patients, achieved mycological eradication at day 14, and were treatment successes based on the 30-day all-cause mortality endpoint. The patient with *C. dubliniensis* had an indeterminate global cure response at day 14, and the patient with *C. glabrata* failed to achieve a global cure response at day 14. The patient with *C. glabrata* had an *FKS* mutant isolate harboring the F659V Fks2 HS1 alteration, while the isolate from the patient with*C. dubliniensis* had no identified mutations in *FKS* HSs.

**TABLE 5:** Outcomes of patients in ReSTORE with baseline *Candida* isolates that were non-susceptible to rezafungin or caspofungin

Patient (treatment group)	Species	Mycological response day 14	Investigator response day 14	Global response day 14	ACM day 30	MIC, µg/mL/CLSI interpretation^*a*^		Fks sequence
						Rezafungin	Caspofungin	
1 (rezafungin)	*C. dubliniensis*	Eradication	Indeterminate	Indeterminate	Alive	0.25/**NS**	0.12/**N/A**	WT
2 (rezafungin)	*C. glabrata*	Eradication	Failure	Failure	Alive	0.5/**S**	0.25/**I**	F659V (Fks2 HS1)

^*a*^ CLSI 2022 (17). ACM, all-cause mortality; HS1, hotspot 1; I, intermediate; N/A, not available; NS, non-susceptible; S, susceptible.

#### Clinical outcomes in patients with post-baseline isolates demonstrating reduced susceptibility to rezafungin or caspofungin

Isolates were recovered from two patients (both from blood cultures) who showed greater than equal to fourfold increases in rezafungin or caspofungin MIC values from baseline. One patient in the rezafungin treatment group had a *C. glabrata* isolate with a rezafungin MIC of 0.06 µg/mL at baseline. Treatment successes for day 5 and day 14 mycological response, day 14 global response, and day 30 all-cause mortality were achieved in this patient. However, at an unscheduled visit on day 35, the *C. glabrata* isolated from this patient had a rezafungin MIC value of 0.5 µg/mL and harbored a mutation in *FKS2* (S663P, Fks2 HS1). The caspofungin MIC value for the isolate at day 35 was also elevated relative to baseline (0.25 vs 0.06 µg/mL, respectively). Based on multilocus sequencing type analysis of whole genome sequence data, both isolates were determined to be type 10, although it is unknown whether this day 35 isolate was isogenic to the *FKS* WT baseline *C. glabrata* or represented infection with a new strain. One patient in the caspofungin treatment group had a *C. albicans* isolate with a rezafungin MIC value of 0.03 µg/mL at baseline, rising to 0.12 µg/mL at day 5, with a concomitant increase in caspofungin MIC values from 0.008 µg/mL (baseline) to 0.06 µg/mL (day 5). Sequencing revealed that both the baseline and day 5 isolate were *FKS1* WT. This patient died and had failed treatment outcomes based on all endpoints: day 5 and day 14 mycological response, day 14 Global Response, and day 30 ACM (i.e., deceased by day 30).

## DISCUSSION

In this analysis of the Phase 3 ReSTORE study, rezafungin demonstrated efficacy based on rates of global cure and mycological eradication at day 14 and the day 30 all-cause mortality rate, regardless of baseline *Candida* spp. Efficacy outcomes did not appear to be impacted by MIC values across *Candida* spp. for either rezafungin or caspofungin. Although the distribution of *Candida* isolates in ReSTORE was largely WT and susceptible to rezafungin based on CLSI interpretation, rezafungin treatment was also successful in the limited number of patients from ReSTORE who had non-susceptible baseline *Candida* isolates and *FKS* mutant isolates.

The MIC data from this study were consistent with the worldwide reported antimicrobial activity (2019–2020) of rezafungin and caspofungin against *Candida* spp. (16). The rates of all-cause mortality at day 30 by pathogen spp. at baseline and treatment-specific MIC value were comparable between the two treatment groups. There were some numerical differences in global cure and mycological eradication between the treatments for certain species; however, the small sample sizes prohibited drawing broad conclusions on comparative efficacy by *Candida* pathogen. In other studies, correlations between MIC values and patient outcomes have not consistently been seen. For example, higher caspofungin MICs correlated with poor treatment outcomes among patients with *C. glabrata*, IC, and prior echinocandin exposure in a retrospective study (19), whereas an analysis of caspofungin clinical trial data found no such correlation (20). In addition, a correlation between high MIC and poor treatment outcomes was found in an analysis of 32 clinical isolates from candidemia patients treated with fluconazole (21) but was not observed in a larger population-based cohort (22). These findings suggest a multifactorial relationship between MIC values and treatment outcomes and the need for additional factors to be considered (23). Catheter placement is one such clinical risk factor affecting treatment outcomes that should be taken into account (24, 25).

A relationship between echinocandin exposure (area under the curve), MIC, and efficacy has been found for micafungin (26), underlining the importance of considering PK/PD parameters when evaluating breakpoints in the context of candidemia and IC. PK/PD simulations of the older echinocandins found that caspofungin and micafungin were likely to achieve therapeutic drug exposures in the majority of simulated patients relative to *C. glabrata* MIC_90_ values, whereas anidulafungin was not likely to achieve therapeutic drug exposures (27). Similar PK/PD simulations with a once-weekly 400 mg rezafungin regimen found a 100% probability of PK/PD target attainment across weeks 1–6 for the *C. glabrata* MIC_90_ of 0.12 µg/mL (28).

A clinical “susceptible” breakpoint for *C. glabrata* of ≤0.5 µg/mL was approved by the CLSI Subcommittee on Antifungal Susceptibility Tests on 20 January 2024; this breakpoint is higher than those established for anidulafungin (≤0.12 µg/mL), caspofungin (≤0.12 µg/mL), and micafungin (≤0.06 µg/mL) (17). As a drug that provides high plasma drug concentrations early in therapy, rezafungin may be better positioned to treat infections caused by isolates with higher MICs, as the epidemiology of *C. glabrata* moves toward reduced susceptibility to treatment with echinocandins. In the rezafungin clinical program, there were three patients (one in the ReSTORE study and two in the expanded access program) who had infections caused by *FKS* mutant *C. glabrata* isolates exhibiting reduced *in vitro* susceptibility to the approved echinocandins and rezafungin; however, all had positive treatment outcomes with rezafungin (29, 30). Of note, the ReSTORE trial *FKS* mutant *C. glabrata* isolate that was a mycological eradication success possessed the same Fks alteration (Fks2 HS1 F659V) as an isolate used in a neutropenic mouse model where rezafungin was also efficacious (11).

Despite a limited sample size, the prevalence of fluconazole-resistant isolates in the ReSTORE trial is reflective of increasing rates observed clinically over time for *C. glabrata*, *C. tropicalis* (particularly in the Asia-Pacific region), and *C. parapsilosis* (8, 9). Notably, all fluconazole-resistant isolates in the trial were susceptible to rezafungin and comparator echinocandins (data not shown), further supporting the role of echinocandins as first-line therapy for candidemia and IC.

As with the primary analysis of the ReSTORE trial (12), potential limitations of this analysis are that the study excluded those with specific forms of IC typically requiring long courses of antifungal treatment or occurring at sites where echinocandin penetration is poor, such as the urinary tract and the central nervous system. The study also excluded pediatric patients. These exclusion criteria limit the generalizability of the results of this analysis to these specific patient subgroups. The relatively small sample size in some of the baseline *Candida* pathogen groups is a further limitation specific to this analysis, as is the small number of samples with elevated and non-WT MICs, and the lack of echinocandin-resistant pathogens and *C. auris* isolates.

### Conclusions

Overall, these data further support the efficacy of rezafungin in candidemia and IC. We found that rezafungin demonstrated efficacy for a global cure, mycological eradication, and day 30 all-cause mortality regardless of baseline *Candida* spp. Efficacy outcomes across *Candida* spp. did not appear to be impacted by MIC values for either rezafungin or caspofungin; assessment of other clinical factors may be warranted.

## MATERIALS AND METHODS

### Study design

ReSTORE (NCT03667690) (12) was a global, double-blind, double-dummy, randomized, Phase 3, non-inferiority trial of rezafungin vs caspofungin. Patients (≥18 years old) with candidemia or IC were randomly assigned (1:1) to receive either rezafungin 400/200 mg intravenously (IV) once weekly or caspofungin 70/50 mg IV once daily for ≥14 days (up to 4 weeks). Patients who met relevant criteria could be switched to oral step-down therapy after ≥3 days of IV therapy (placebo for the rezafungin group and fluconazole for the caspofungin group). The study was performed at 66 tertiary care centers across 15 countries between October 2018 and October 2021. The study was performed in compliance with the International Conference on Harmonisation Good Clinical Practice and the Declaration of Helsinki. All patients provided written informed consent. For full study details, please refer to the primary publication (12).

### Eligibility criteria

Key inclusion criteria were an established mycological diagnosis of candidemia and/or IC ≤96 hours before randomization (≥1 blood culture positive for yeast or *Candida*, a positive test for *Candida* from a rapid *in vitro* diagnostic test, or a positive Gram stain for yeast or a positive culture for *Candida* spp. from a specimen obtained from a normally sterile site) and ≥1 systemic signs attributable to candidemia or IC appearing from ≤12 hours prior to the qualifying positive culture through to the time of randomization. If the positive blood culture was drawn >12 hours prior to randomization, an additional set of blood cultures was obtained≤12 hours before randomization to confirm *Candida* spp. status at enrollment. Results of the blood cultures obtained ≤12 hours before randomization were not required for the patient to be enrolled in the study. Patients with prosthetic joint septic arthritis, osteomyelitis, endocarditis, myocarditis, meningitis, endophthalmitis, central nervous system infection, chronic disseminated candidiasis, or urinary tract candidiasis were excluded, as were patients who had received systemic treatment with an antifungal agent for >48 hours in the 96 hours before randomization or who had an indwelling vascular catheter/device that could not be removed and that was likely to be the source of candidemia.

### Assessments and outcomes

This analysis assessed outcomes among patients treated with rezafungin or caspofungin in the ReSTORE mITT in subgroups defined by *Candida* spp. and *in vitro* susceptibility at baseline. The mITT population was defined as patients who had a documented *Candida* infection based on central laboratory evaluation of culture from blood or another normally sterile site obtained ≤4 days (96 hours) before randomization and who received ≥1 dose of the study drug.

The genus and spp. of *Candida* pathogens were identified using mass spectrometry (matrix-assisted laser desorption/ionization–time of flight mass spectrometry, Bruker Daltonics, Bremen, Germany) by central laboratory evaluation of baseline blood and sterile site cultures. For susceptibility testing, CLSI (31) broth microdilution methodology was used to determine MIC values to rezafungin, caspofungin, anidulafungin, micafungin, and fluconazole for each *Candida* spp. Susceptibility interpretations for rezafungin and caspofungin were determined using the CLSI breakpoints (17) and those recently granted for rezafungin by the FDA (18).

Echinocandin non-susceptible isolates were submitted for whole-genome sequencing to screen for *FKS* mutations. Total genomic DNA was used as the input material for the library, which was sequenced using a MiSeq Sequencer (Illumina, San Diego, CA, USA). Reads were trimmed with Sickle, version 1.33 (32), with the error corrected using BayesHammer (33) in SPAdes 3.11.1 (34). Each sample was assembled using a reference-guided assembly in SeqMan NGen, version 16.0 (DNASTAR, Madison, WI, USA). DNA regions encoding *FKS* genes were compared to sequences available in the literature (35).

ReSTORE included two different primary efficacy endpoints; one (global cure at day 14) required by the European Medicines Agency and one (all-cause mortality at day 30) by the FDA. Global cure (defined as achieving clinical cure, mycological eradication, and, for patients with IC diagnosed by radiology, radiological cure; all confirmed by the independent data review committee) was assessed at day 14 (±1 day); and all-cause mortality was evaluated through day 30. Mycological response (eradication, failure, or indeterminate) was assessed at day 14 (±1 day) and at other timepoints; day 14 results are presented here unless otherwise stated. Mycological eradication was defined in these patients as a negative post-baseline blood culture or a negative post-baseline culture from another normally sterile site. If a follow-up culture from the normally sterile site other than blood was not accessible, the patient had to have achieved a successful clinical outcome, as assessed by the investigator, without a change of antifungal therapy for the treatment of candidemia and/or IC.

For this analysis, only descriptive data are presented, as counts and percentages.

## SUPPLEMENTAL MATERIAL

Supplementary Tables aac.01584-23-s0001.pdf Table S1, Table S2.

ASM does not own the copyrights to Supplemental Material that may be linked to, or accessed through, an article. The authors have granted ASM a non-exclusive, world-wide license to publish the Supplemental Material files. Please contact the corresponding author directly for reuse.
