# Supplementary material for: Outcomes by Candida spp. in the ReSTORE Phase 3 trial of rezafungin versus caspofungin for candidemia and/or invasive candidiasis
Source: Antimicrob Agents Chemother. 2024 Mar 25;68(5):e01584-23. doi: 10.1128/aac.01584-23 (PMC11064504; doi:10.1128/aac.01584-23)
Supplement: Supplementary Tables — Table S1, Table S2. [file aac.01584-23-s0001.pdf]

SUPPLEMENTAL MATERIAL

Table S1 Antimicrobial activity of rezafungin and caspofungin against baseline *Candida* species[a]

Organism [no. of isolates]	Number (cumulative %) of isolates inhibited at an MIC (µg/mL) of:												
	**0.004**	**0.008**	**0.015**	**0.03**	**0.06**	**0.12**	**0.25**	**0.5**	**1**	**2**	**MIC_{50}**	**MIC_{90}**	**MIC range**
**Rezafungin**													
C. albicans [78]		7 (17.9)	20 (69.2)	6 (84.6)	4 (94.9)	2 (100)					0.015	0.06	0.008–0.12
C. glabrata [49]				8 (33.3)	6 (58.3)	9 (95.8)		1 (100)			0.06	0.12	0.03–0.5
C. tropicalis [36]			3 (15.0)	8 (55.0)	7 (90.0)	2 (100)					0.03	0.06	0.015–0.12
C. parapsilosis [24]								1 (12.5)	4 (62.5)	3 (100)	–	–	0.5–2
**Caspofungin**													
C. albicans [78]		2 (5.1)	9 (28.2)	21 (82.1)	6 (97.4)	1 (100)					0.03	0.06	0.008–0.12

Organism [no. of isolates]	Number (cumulative %) of isolates inhibited at an MIC (µg/mL) of:										MIC$_{50}$	MIC$_{90}$	MIC range
	0.004	0.008	0.015	0.03	0.06	0.12	0.25	0.5	1	2			
C. glabrata [49]				5 (20.0)	19 (96.0)	1 (100)					0.06	0.06	0.03–0.12
C. tropicalis [36]			1 (6.3)	7 (50.0)	7 (93.8)	1 (100)					0.03	0.06	0.015–0.12
C. parapsilosis [24]							8 (50.0)	8 (100)			0.25	0.5	0.25–0.5

[a]For patients with multiple specimens having the same baseline spp. tested, the specimen with the highest MIC to the study drug received was used. Only spp. isolated at least 10 times in a treatment group are included here. MIC$_{50/90}$ are provided for pathogens isolated at least 10 times in a treatment group.

MIC$_{50/90}$, minimum inhibitory concentration required to inhibit 50% / 90% of isolates tested, respectively.

Table S2 Day-30 all-cause mortality by baseline *Candida* spp. and rezafungin and caspofungin CLSI MIC values

***Candida* spp.** Treatment [isolates[b]]	**n/N (%) by treatment-specific MIC value, μg/mL[a]**								
	**0.008**	**0.015**	**0.03**	**0.06**	**0.12**	**0.25**	**0.5**	**1**	**2**
C. albicans									
Rezafungin [39]	1/7 (14.3)	6/20 (30.0)	1/6 (16.7)	2/4 (50.0)	1/2 (50.0)				
Caspofungin [39]	1/2 (50.0)	3/9 (33.3)	3/21 (14.3)	1/6 (16.7)	1/1 (100)				
C. glabrata									
Rezafungin [24]			1/8 (12.5)	1/6 (16.7)	2/9 (22.2)		0/1 (0)		
Caspofungin [25]			0/5 (0)	2/19 (10.5)	0/1 (0)				
C. tropicalis									
Rezafungin [20]		0/3 (0)	2/8 (25.0)	2/7 (28.6)	1/2 (50.0)				
Caspofungin [16]		0/1 (0)	3/7 (42.9)	1/7 (14.3)	0/1 (0)				
C. parapsilosis									
Rezafungin [8]							0/1 (0)	1/4 (25.0)	0/3 (0)
Caspofungin [16]						3/8 (37.5)	2/8 (25.0)		

[a]n/N is the number of patients with the corresponding *Candida* pathogen who died on or before Day 30 or with unknown survival status/number of patients with the corresponding *Candida* pathogen and MIC value at baseline; not all isolates had MIC data; [b]Number of isolates.

CLSI, Clinical and Laboratory Standards Institute.
